# Acute Effects of Static Stretching Duration on a Single-Leg Balance Task

**DOI:** 10.3390/sports13060188

**Published:** 2025-06-18

**Authors:** Takamasa Mizuno

**Affiliations:** Research Center of Health, Physical Fitness and Sports, Nagoya University, Nagoya-shi 464-8601, Aichi, Japan; mizuno@htc.nagoya-u.ac.jp; Tel.: +81-52-789-3959

**Keywords:** flexibility, ultrasound, passive torque, center of pressure, plantar fascia

## Abstract

The purpose of this study was to determine the effect of static stretching (SS) duration on balance. Twenty-two participants performed passive dorsiflexion measurements and balance tests before and after SS. Passive dorsiflexion measurements determined the maximal dorsiflexion angle, passive torque, displacement of the muscle–tendon junction, and electromyography amplitude during passive dorsiflexion. In the balance test, the participant stood on a single leg with their eyes open while the postural sway evaluated in the center of pressure (COP), standing duration, and electromyography amplitude were measured. The ankle and metatarsophalangeal joints underwent SS for 30 s × one set, two sets, and four sets. There were significant increases in COP displacement and COP velocity after two sets of SS but not after one and four sets. Standing duration and electromyography during balance tests were not changed after SS. No gender differences were found in changes in balance. Maximal dorsiflexion angle and passive torque were increased after SS, but the displacement of the muscle–tendon junction and electromyography during passive dorsiflexion were not changed. There was no significant correlation between changes in maximal dorsiflexion angle or passive torque and changes in COP variables after two sets of SS. These results therefore revealed that SS duration affects COP displacement and COP velocity.

## 1. Introduction

Maintaining balance means maintaining the center of pressure (COP) within a changing base plane of support [1]. Balance is an important factor for many populations because of its reported associations with sports performance [2] and functional activity in older adults [3]. In addition, impaired balance increases the risk of injuries [4] and falls in the elderly population [5]. Therefore, it is essential to maintain and improve balance throughout life. Balance control is related to somatomotor perception and muscular strength [6,7]. Hence, it has been shown that balance may be affected by static stretching (SS), which reduces muscle strength and stiffness and causes changes in intrinsic sensory and neural properties [8,9,10,11,12,13].

Joint flexibility properties have also been shown to affect balance [14,15]. A previous study showed an association between hip and ankle active range of motion and the Y balance test score [14]. Another study reported a significant correlation between passive torque at the maximal dorsiflexion angle during passive ankle dorsiflexion and the COP area [15]. In the elderly, a decreased range of motion has been suggested to be associated with an increased risk of falls [16]. Therefore, joint flexibility properties are important factors in maintaining and improving balance. However, flexibility properties such as range of motion, passive torque, stiffness, stretch tolerance, and displacement of the muscle–tendon junction are changed after SS [17]. One study reported a significant correlation between a change in stiffness of the muscle–tendon unit after SS and a change in balance after SS in elderly men [18]. However, it is unclear whether there is an association between changes in flexibility properties after SS and balance after SS when the individual being assessed is a young adult, when the measurement site is the lower leg, or when balance is assessed using COP variables.

Previous studies have reported conflicting results regarding the acute effects of SS on balance [11,19,20]; it has been shown to improve [19,20,21,22,23,24,25,26], impair [9,10,11,21,27], and not change [19,28,29,30,31,32] balance. In addition, a review article stated that further research is needed because there are a similar number of studies reporting positive and negative effects of SS on balance [33], whereas another meta-analysis reported that acute SS had no significant effect on static standing sway/COP variables when assessed with the eyes open [34]. These discrepancies are assumed to be caused by differences in stretching methods (e.g., the stretching intensity, duration, and site) and balance assessment methods (e.g., static or dynamic balance, open or closed eyes, and assessment by COP variables or by unique balance index). In particular, the effect of stretching duration on balance is unclear. Among the factors affecting balance, muscle strength, range of motion, stiffness, and proprioception have been reported to change after SS, and the amount of change in these variables is influenced by the stretching duration [35,36,37,38]. In addition, deficits in balance after SS have been explained by SS-induced deficits in muscle strength and stiffness [9,34]; however, these are not seen when SS is carried out for a shorter duration, and significant changes are shown with increasing SS duration [36]. Therefore, it is important to systematically assess the effect of SS duration on balance, in addition to practical SS duration [33]. Thus, it was hypothesized that balance would be impaired with increasing SS duration. It was also hypothesized that the stretching-induced decrease in passive torque, an index that assesses the mechanical properties of muscle–tendon unit, as well as stiffness, would be associated with impaired balance assessed after SS using COP variables. To clarify these hypotheses, the purpose of this study was to determine the acute effects of SS duration on balance. This study also examined whether gender affected the effect of static stretching time on balance.

## 2. Materials and Methods

### 2.1. Study Participants

The number of participants required was calculated using the following parameters: power = 0.80, α = 0.05, effect size = 0.19 (calculated from Oba et al. [30]); this indicated that 20 participants were needed (G*Power 3.1). Twenty-two adults (10 men, age: 20.6 ± 1.2 years, height: 172.1 ± 9.0 cm, weight: 60.0 ± 8.6 kg; 12 women, age: 20.3 ± 2.5 years, height: 159.1 ± 9.2 cm, weight: 50.9 ± 12.0 kg) participated in the experiment. Fourteen participants had no recent exercise habits, while four men and four women reported jogging, walking, or playing badminton for 6.2 ± 3.7 h per week. No one practiced exercise competitively; it was performed as a recreational activity. None of the participants had a history of recent musculoskeletal injury or neuromuscular disease specific to the lower leg. All participants provided written informed consent to participate in the experiments, which were conducted in accordance with the principles of the Declaration of Helsinki. All participants were fully informed of the purpose, procedures, and potential risks of the study. The experimental protocol was approved by the Ethics Committee of Nagoya University (23-01).

### 2.2. Experimental Design

**Figure 1:**
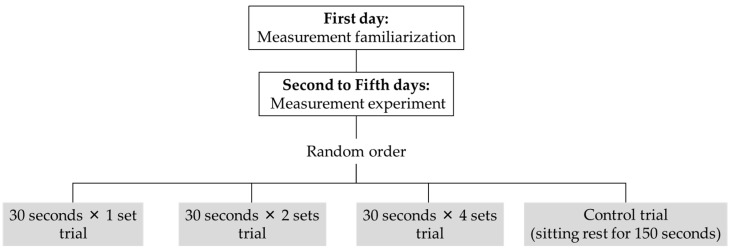
Experimental design.

Each participant visited the laboratory five times (Figure 1). The first session consisted of the researcher explaining the experiment and obtaining consent and the participant undertaking a familiarization trial of measurements and stretching. Data were collected in the second through fifth sessions. After warming up on the bicycle ergometer (50 w × 3 min), passive dorsiflexion measurements and balance tests were performed. The passive dorsiflexion measurements and balance tests were then repeated after SS (30 s × 1, 2, or 4 sets) or sitting rest for 150 s (CON). Measurements were taken on different days for each stretching trial or CON. The order in which the passive dorsiflexion measurements and balance tests were performed was determined randomly, but the order was same between sessions for a given volunteer. The order of SS durations was also determined randomly.

### 2.3. Passive Dorsiflexion Measurement

**Figure 2:**
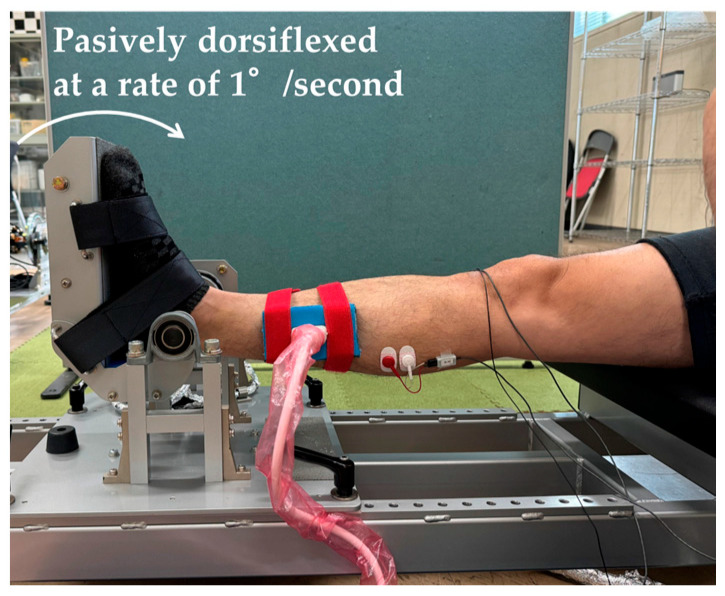
Passive dorsiflexion measurement.

The ankle angle, passive torque, and displacement of the muscle–tendon junction were determined in the passive dorsiflexion measurements, which were performed in the same way as reported in a previous study [17]. The participant sat on the isokinetic machine (S-15177; Takei Scientific Instruments, Niigata, Japan) with the right ankle joint fixed to the footplate and the right knee fully extended (Figure 2). The ankle joint was passively dorsiflexed by the footplate at a rate of 1°/second from the plantar flexion position to the maximal dorsiflexion angle. The maximal dorsiflexion angle was defined as the angle at which the participant first felt pain in the lower leg due to dorsiflexion. The participant stopped dorsiflexion by pressing a button (safety switch) when the pain sensation occurred. The footplate angle was shown as the ankle joint angle, which was defined as 0° when the footplate was perpendicular to the floor. Dorsiflexion was indicated by positive values. Passive torque was assessed at the maximal dorsiflexion angle and during the final 13° period common to pre-SS and post-SS measurements during each trial for each participant [38]. The passive torque was evaluated every 4° during the final 13° of dorsiflexion [38].

The displacement of the muscle–tendon junction of the gastrocnemius medialis during passive ankle dorsiflexion was determined using B-mode ultrasonography (Versana Active; GE Healthcare Japan, Tokyo, Japan). A linear array probe (12L-RS; GE Healthcare Japan, Tokyo, Japan) was fixed to the skin. The muscle–tendon junction was visualized on a longitudinal ultrasound image and synchronized with the passive torque and ankle angle output. The relative displacement between the reflective marker affixed to the skin and the muscle–tendon junction was measured. Displacement of the muscle–tendon junction was analyzed using the open license software Kinovea (version 0.8.27). The displacement of the muscle–tendon junction was evaluated at the maximal dorsiflexion angle and during the final 13° of dorsiflexion, the same as the passive torque assessment. Passive dorsiflexion measurements were collected twice before SS and once after SS; for the pre-SS measurements, data from the measurement with the larger maximal dorsiflexion angle out of the two measurements were used for subsequent analysis.

### 2.4. Balance Test

The balance test measured the standing duration and COP variables during single-leg standing with the eyes opened. The participants were instructed to stand on the measuring device so that the ball of the right foot was in line with the center of the device (T.K.K.5810; Takei Scientific Instruments, Niigata, Japan). Participants were instructed to place their hands on their waist and then float the sole of their left foot 5–10 cm above the device with their eyes open and looking forward for a maximum of 20 s. The measurement time of the balance test was identical to that of the previous study [23]. If the left foot reached the measuring device, a hand left the waist, or the right foot was displaced before 20 s were reached, the measurement was terminated at that point. The COP was analyzed using data from participants who reached 20 s in both the pre-SS and post-SS balance tests. The COP was analyzed using 10 of the 20 s of data, after excluding the first and last 5 s of each measurement [23]. COP was evaluated using displacement, velocity, and area as variables. Balance tests were conducted twice pre-SS and once post-SS; for the pre-SS tests, data from the test with the longer standing duration out of the two tests were used for subsequent analysis. If the standing duration between the pre-tests was the same, the shorter COP displacement out of the two tests was used for subsequent analysis.

### 2.5. Electromyography

Muscle activity during passive dorsiflexion measurements and balance tests was assessed using electromyography (EMG) (DL-140; S&ME, Tokyo, Japan). Disposable surface electrodes (F-150s; Nihon Kohden, Tokyo, Japan) were applied to the gastrocnemius medialis and tibialis anterior muscles with a distance of 20 mm between electrodes. Electrodes were positioned at the most prominent bulge of the gastrocnemius medialis muscle and one-third of the distance from the tip of the fibula to the medial ankle, in accordance with the Surface EMG for Noninvasive Assessment of Muscles guidelines. The reference electrode was placed on the fibular head. EMG signals were sent to a digital data recorder at a sampling rate of 1.0 kHz and recorded with a bandwidth of 20–500 Hz. In the passive dorsiflexion test, the root mean square was calculated from 30° of plantar flexion to 25° of plantar flexion (initial period) and for the last 5° interval from an angle 5° less than the maximal dorsiflexion position to the maximal dorsiflexion position (final period). In the balance test, the root mean square was calculated for 10 of the 20 s of data, after excluding the first and last 5 s. EMG amplitudes were normalized to allow for inter-participant comparisons. One maximal isometric plantar flexion contraction at 0° for 5 s and one maximal isometric dorsiflexion contraction at 0° for 5 s were performed after conducting post measurements. The root mean square data in passive dorsiflexion measurement and balance test were normalized by the peak EMG amplitude during maximal isometric contraction for each muscle.

### 2.6. Static Stretching

**Figure 3:**
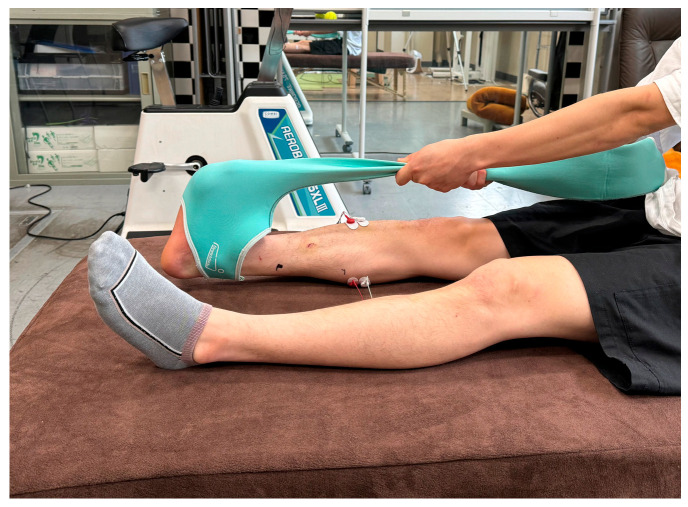
Static stretching using a self-stretching aid item.

The plantar flexors and plantar fascia underwent SS using a self-stretching aid item (Stretch Sox; YOKOYAMA SEIMITSU Co., Ltd., Hiroshima, Japan). The participants wore Stretch Sox on their right foot and used their hands to pull it to dorsiflex the ankle and metatarsophalangeal joints (Figure 3). The stretching duration was 30 s × 1 set, 2 sets, or 4 sets, with a 10 s rest between sets. The stretching intensity was defined as the angle at which the participant felt the maximal lengthening sensation without pain in the area being stretched.

### 2.7. Measurement Reproducibility

Test–retest reliability for two consecutive measurements is shown using the intra-class correlation coefficient. The reproducibility of the passive dorsiflexion measurement was already reported in the author’s previous study using the same measurement method as in the present study [39], with the maximal dorsiflexion angle of 0.967, the passive torque at a maximal dorsiflexed position of 0.965, and the displacement of the muscle–tendon junction at a maximal dorsiflexed position of 0.841. The repeatability of the balance test was evaluated by calculating the intra-class correlation coefficient using the data from the two obtained in the pre-measurement, with a COP displacement of 0.759.

### 2.8. Statistical Analysis

In the balance test, 20 participants (9 men and 11 women) achieved 20 s of open-eyed single-leg standing in all measurements. Therefore, the COP variables were analyzed using data from these 20 participants. Due to equipment malfunction, the EMG during maximal isometric muscle contraction could not be measured in three participants. Therefore, these 3 participants were excluded from the EMG analysis, which therefore analyzed EMG data from 17 participants. Other measurement data were analyzed with data from 22 participants. All statistical analyses were performed using SPSS version 22.0 (IBM Corp., Armonk, NY, USA). Three-way analysis of variance (ANOVA; time (pre- or post-stretching) × trial (1 set, 2 sets, 4 sets, or CON) × angle (final 1°, final 5°, final 9°, final 13°, or maximal dorsiflexed position)) was used to analyze the passive torque and the displacement of the muscle–tendon junction. Three-way ANOVA (time (pre- or post-stretching) × trial (1 set, 2 sets, 4 sets, or CON) × period (initial or final]) was used to analyze the EMG amplitude during passive dorsiflexion measurements. Three-way ANOVA (time (pre- or post-stretching) × trial (1 set, 2 sets, 4 sets, or CON) × gender (man or woman)) was used to analyze the gender difference in single-leg standing duration, EMG amplitude during balance test, and COP displacement, velocity, and area. Analyses of the maximal dorsiflexion angle, single-leg standing duration, EMG amplitude during balance test, and COP displacement, velocity, and area were conducted using two-way ANOVA (time (pre- or post-stretching) × trial (1 set, 2 sets, 4 sets, or CON)). Partial eta squared (*η_p_*^2^) values were calculated from the main effects or interactions in the repeated-measures ANOVA, with values of 0.01, 0.06 and above 0.14 representing small, medium and large differences, respectively [40]. Follow-up analyses were performed using lower-order ANOVA and t-tests with Bonferroni correction. Effect sizes were calculated for pair-wise comparison using Cohen’s d, defined as small (d < 0.4), moderate (0.41 < d < 0.7), or large (0.8 < d) magnitudes of change [40]. In trials where there was a significant change in the COP variable after SS, Pearson’s correlation coefficient was used to analyze the relationship between the change in the maximal dorsiflexion angle or passive torque and the changes in COP variables. Statistical significance was set at *p* ≤ 0.05. All data are reported as mean ± standard deviation.

## 3. Results

### 3.1. Balance Test

#### 3.1.1. Single-Leg Standing Duration

No significant two-way interaction (*η_p_*^2^ = 0.034) and no significant main effects were identified for time (*η_p_*^2^ = 0.080) or trial (*η_p_*^2^ = 0.034) (one set: pre-SS 20.0 ± 0.0 s, post-SS 20.0 ± 0.0 s; two sets: pre-SS 20.0 ± 0.0 s, post-SS 19.4 ± 2.7 s; four sets: pre-SS 20.0 ± 0.0 s, post-SS 20.0 ± 0.0 s; CON: pre-SS 20.0 ± 0.0 s, post-SS 19.7 ± 1.2 s). The single-leg standing duration with eyes opened was not changed after SS or CON.

#### 3.1.2. Center of Pressure Displacement

**Figure 4:**
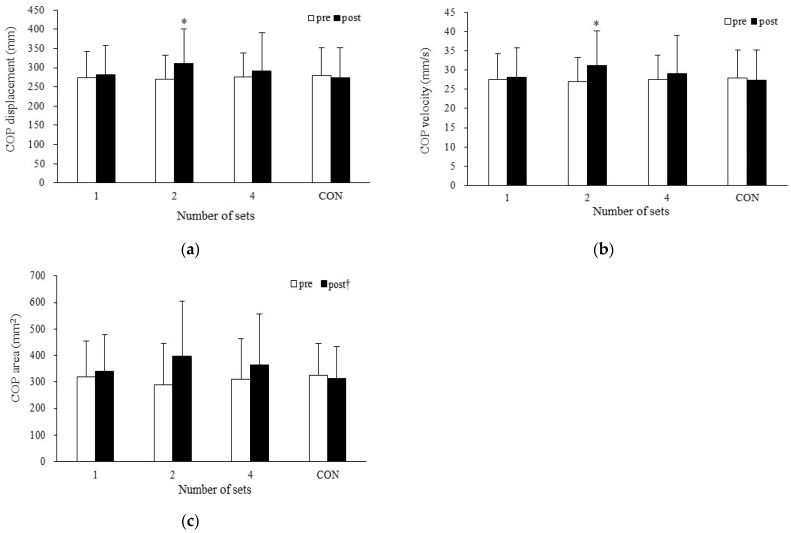
SS-induced changes in (**a**) COP displacement, (**b**) COP velocity, and (**c**) COP area. Data are expressed as mean ± standard deviation. * Significantly different from pre-SS (*p* < 0.05). † Significant difference from pre-SS to post-SS COP area collapsed across number of sets (*p* < 0.05). SS: static stretching; COP: center of pressure; CON: control.

A significant two-way interaction was noted (*p* = 0.035, *η_p_*^2^ = 0.139) (one set: pre-SS 274.7 ± 67.1 mm, post-SS 281.9 ± 76.3 mm; two sets: pre-SS 269.7 ± 63.0 mm, post-SS 311.4 ± 90.0 mm; four sets: pre-SS 276.3 ± 62.8 mm, post-SS 291.0 ± 99.9 mm; CON: pre-SS 279.2 ± 73.0 mm, post-SS 273.6 ± 78.5 mm). Post-hoc testing revealed that the COP displacement was increased after two sets of SS (*p* = 0.003, *d* = 0.66) (Figure 4a).

#### 3.1.3. Center of Pressure Velocity

A significant two-way interaction was noted (*p* = 0.035, *η_p_*^2^ = 0.139) (one set: pre-SS 27.5 ± 6.7 mm/s, post-SS 28.2 ± 7.6 mm/s; two sets: pre-SS 27.0 ± 6.3 mm/s, post-SS 31.1 ± 9.0 mm/s; four sets: pre-SS 27.6 ± 6.3 mm/s, post-SS 29.1 ± 10.0 mm/s; CON: pre-SS 27.9 ± 7.3 mm/s, post-SS 27.4 ± 7.8 mm/s). Post-hoc testing revealed that the COP velocity was increased after two sets of SS (*p* = 0.003, *d* = 0.66) (Figure 4b).

#### 3.1.4. Center of Pressure Area

There was no significant two-way interaction (*η_p_*^2^ = 0.089) and no significant main effect for trial (*η_p_*^2^ = 0.012). However, a significant main effect was identified for time (*p* = 0.032, *η_p_*^2^ = 0.220) (one set: pre-SS 319.6 ± 133.6 mm^2^, post-SS 340.8 ± 137.2 mm^2^; two sets: pre-SS 290.3 ± 155.7 mm^2^, post-SS 395.6 ± 209.0 mm^2^; four sets: pre-SS 310.0 ± 154.7 mm^2^, post-SS 365.3 ± 192.1 mm^2^; CON: pre-SS 323.5 ± 120.9 mm^2^, post-SS 311.5 ± 122.0 mm^2^). Post-hoc testing revealed that the COP area was increased from pre-SS to post-SS (*d* = 0.44) (Figure 4c).

#### 3.1.5. Electromyography Amplitude in the Balance Test

There were no significant two-way interactions (*η_p_*^2^ = 0.055) and no significant main effects for time (*η_p_*^2^ = 0.007) or trial (*η_p_*^2^ = 0.027) in the gastrocnemius medialis. Similarly, there were no significant two-way interactions (*η_p_*^2^ = 0.103) and no significant main effects for time (*η_p_*^2^ = 0.088) or trial (*η_p_*^2^ = 0.017) in the tibialis anterior muscles. There were no significant changes in the EMG amplitude after SS or CON (Table 1).

### 3.2. Gender Difference in Balance Test

#### 3.2.1. Single-Leg Standing Duration

No significant three-way interaction (*η_p_*^2^ = 0.054) and no two-way interactions for time and trial (*η_p_*^2^ = 0.031), trial and gender (*η_p_*^2^ = 0.054), or time and gender (*η_p_*^2^ = 0.007) were identified. In addition, no significant main effects were identified for time (*η_p_*^2^ = 0.076), gender (*η_p_*^2^ = 0.007), or trial (*η_p_*^2^ = 0.031). No gender differences were found in the change in single-leg standing duration with eyes opened after SS or CON (Table 2).

#### 3.2.2. Center of Pressure Displacement

No significant three-way interaction (*η_p_*^2^ = 0.051) and no two-way interactions for trial and gender (*η_p_*^2^ = 0.034) or time and gender (*η_p_*^2^ = 0.069) were identified. However, a significant two-way interaction was noted for time and trial (*p* = 0.025, *η_p_*^2^ = 0.157). Post-hoc testing revealed that the COP displacement was increased after two sets of SS (*p* = 0.002, *d* = 0.52). However, no gender differences were found in the change in COP displacement after SS or CON (Table 2).

#### 3.2.3. Center of Pressure Velocity

No significant three-way interaction (*η_p_*^2^ = 0.051) and no two-way interactions for trial and gender (*η_p_*^2^ = 0.034) or time and gender (*η_p_*^2^ = 0.070) were identified. However, a significant two-way interaction was noted for time and trial (*p* = 0.025, *η_p_*^2^ = 0.158). Post-hoc testing revealed that the COP velocity was increased after two sets of SS (*p* = 0.002, *d* = 0.52). However, no gender differences were found in the change in COP velocity after SS or CON (Table 2).

#### 3.2.4. Center of Pressure Area

No significant three-way interaction (*η_p_*^2^ = 0.088) and no two-way interactions for time and trial (*η_p_*^2^ = 0.113), trial and gender (*η_p_*^2^ = 0.016), or time and gender (*η_p_*^2^ = 0.145), and no main effect for trial (*η_p_*^2^ = 0.013), were identified. However, significant main effects were identified for time (*p* = 0.018, *η_p_*^2^ = 0.273) and gender (*p* = 0.050, *η_p_*^2^ = 0.196). Post-hoc testing revealed that the COP area was increased from pre-SS to post-SS (*d* = 0.43). Additionally, COP area was greater in men than women (*d* = 0.94). However, no gender differences were found in the change in COP area after SS or CON (Table 2).

#### 3.2.5. Electromyography Amplitude in the Balance Test

No significant three-way interaction (*η_p_*^2^ = 0.090) and no two-way interactions for time and trial (*η_p_*^2^ = 0.064), trial and gender (*η_p_*^2^ = 0.076), or time and gender (*η_p_*^2^ = 0.067) were identified. In addition, no significant main effects were identified for time (*η_p_*^2^ = 0.011), gender (*η_p_*^2^ = 0.160), or trial (*η_p_*^2^ = 0.035) in the gastrocnemius medialis. Similarly, no significant three-way interaction (*η_p_*^2^ = 0.137) and no two-way interactions for time and trial (*η_p_*^2^ = 0.109), trial and gender (*η_p_*^2^ = 0.150), or time and gender (*η_p_*^2^ = 0.154) were identified. In addition, no significant main effects were identified for time (*η_p_*^2^ = 0.131), gender (*η_p_*^2^ = 0.041), or trial (*η_p_*^2^ = 0.036) in the tibialis anterior muscles. No gender differences were found in the change in EMG amplitude after SS or CON (Table 2).

### 3.3. Passive Dorsiflexion Test

#### 3.3.1. Maximal Dorsiflexion Angle

**Figure 5:**
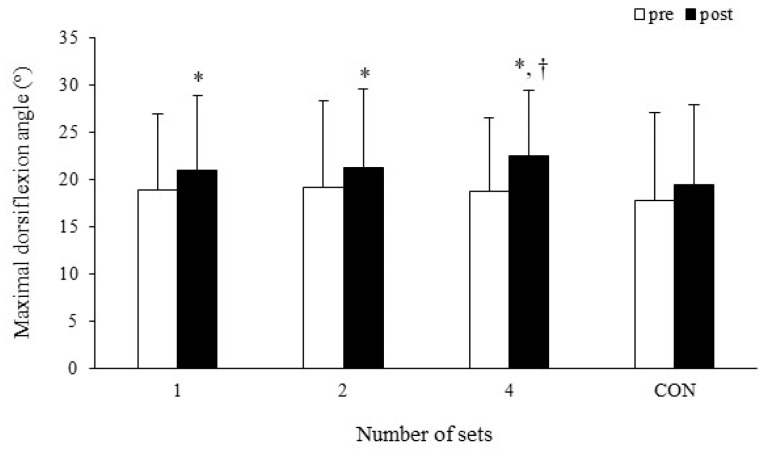
Stretching-induced changes in maximal dorsiflexion angle. Data are expressed as mean ± SD. * Significantly different from pre (*p* < 0.05). † Significantly different from post of control (*p* < 0.05). CON: control.

Two-way ANOVA showed a significant interaction (*p* = 0.011, *η_p_*^2^ = 0.162). The results of the post-hoc test showed that the maximal dorsiflexion angle was significantly increased after one set (*p* < 0.001, *d* = 0.26), two sets (*p* = 0.016, *d* = 0.22), and four sets (*p* < 0.001, *d* = 0.47) of SS, but not after CON (*p* = 0.148, *d* = 0.11) (one set: pre-SS 18.9 ± 8.0°, post-SS 21.0 ± 7.9°; two sets: pre-SS 19.2 ± 9.2°, post-SS 21.2 ± 8.3°; four sets: pre-SS 18.7 ± 7.8°, post-SS 22.5 ± 7.0°; CON: pre-SS 17.8 ± 9.2°, post-SS 19.4 ± 8.5°). In addition, the maximal dorsiflexion angle was larger after four sets of SS than after the CON condition (*p* = 0.012, *d* = 0.36) (Figure 5).

#### 3.3.2. Passive Torque

A significant three-way interaction was noted (*p* = 0.033, *η_p_*^2^ = 0.135). Post-hoc testing revealed that the passive torque was increased from pre-SS to post-SS at the final 1° for two sets (*p* = 0.002, *d* = 0.12), four sets (*p* = 0.023, *d* = 0.09), and CON (*p* = 0.037, *d* = 0.07); at the final 5° for two sets (*p* = 0.001, *d* = 0.10) and four sets (*p* = 0.041, *d* = 0.18); and at the maximal dorsiflexed position for one set (*p* = 0.006, *d* = 0.23), two sets (*p* = 0.011, *d* = 0.24), and four sets (*p* = 0.002, *d* = 0.41). In addition, the passive torque at the maximal dorsiflexed position was greater after one set of post-SS than after the CON condition (*p* = 0.036, *d* = 0.17) (Table 3).

#### 3.3.3. Displacement of the Muscle–Tendon Junction

There was no significant three-way interaction (*η_p_*^2^ = 0.042) and no significant two-way interactions between time and trial (*η_p_*^2^ = 0.010) or between trial and angle (*η_p_*^2^ = 0.035), but there was a significant two-way interaction between time and angle (*p* = 0.006, *η_p_*^2^ = 0.212). Post-hoc testing revealed that the displacement of the muscle–tendon junction increased as the angle increased (all *p* < 0.001), except for the difference between the final 13° of dorsiflexion and the maximal dorsiflexed position before SS (Table 4).

#### 3.3.4. Electromyography Amplitude in Passive Dorsiflexion Measurement

There were no significant three-way interactions (*η_p_*^2^ = 0.066); no significant two-way interactions for time and period (*η_p_*^2^ = 0.043), time and trial (*η_p_*^2^ = 0.038), or trial and period (*η_p_*^2^ = 0.050); and no significant main effects for time (*η_p_*^2^ = 0.082), trial (*η_p_*^2^ = 0.019), or period (*η_p_*^2^ = 0.134) in the gastrocnemius medialis. Similarly, there were no significant three-way interactions (*η_p_*^2^ = 0.024); no significant two-way interactions for time and period (*η_p_*^2^ = 0.009), time and trial (*η_p_*^2^ = 0.004), or trial and period (*η_p_*^2^ = 0.063); and no significant main effects for time (*η_p_*^2^ = 0.001), trial (*η_p_*^2^ = 0.042), or period (*η_p_*^2^ = 0.066) in the tibialis anterior muscles. There were no significant changes in the EMG amplitude after SS or the CON condition (Table 5).

### 3.4. Correlation Coefficient

Correlation coefficients were evaluated for two sets of the SS trial that showed changes in COP variables after SS. There was no significant correlation coefficient of the relationship between the change in maximal dorsiflexion angle or passive torque after SS and the change in COP variables after SS (Table 6).

## 4. Discussion

The purpose of the present study was to determine the effect of SS duration on balance. The COP displacement and COP velocity were increased after two sets of SS but not after one and four sets of SS. There were no gender differences in SS-induced changes in standing duration, COP valuables, or EMG amplitude in the balance test. In addition, no significant correlation was observed between the change in maximal dorsiflexion angle or passive torque and the changes in the COP variables after two sets of SS. Therefore, the present study clarified that the changes in balance after SS were influenced by the SS duration. However, there was no significant relationship between changes in joint flexibility and changes in balance after SS.

The present study found that the effect of SS on balance was dependent on the SS duration. The finding that 30 s of SS had no effect on balance is consistent with previous research [29]. Lim et al. [29] reported that SS for 30 s does not change mediolateral or anteroposterior postural sway. However, two 30 s sets of SS impaired balance in the present study. Similarly, some previous studies have also reported that SS impairs balance [9,10,11]. SS-induced balance deficits have been explained as being due to SS-induced muscle fatigue or SS-induced muscle strength deficits [34,41]. SS-induced muscle strength deficits are more likely to be induced by SS performed for more than 60 s [36,42]. Thus, it is possible that muscle strength deficits occurred after two 30 s sets of SS in the present study, resulting in impaired balance. Another suggested mechanism of balance impairment is a decrease in the stiffness of the muscle–tendon unit [9]. It has been suggested that a stretching-induced decrease in stiffness causes more slack in series and parallel elastic components, thus affecting both the system afferent inputs of muscles to the central nervous system and the output of muscles to balance against unexpected disturbances [9]. However, in the present study, the passive torque during the final 13° of dorsiflexion was not decreased after stretching, suggesting that there was no decrease in stiffness.

The most interesting finding of the present study was that there was no change in balance after four sets of SS, while balance was impaired after two sets of SS. This finding may be explained by changes in the stiffness of the plantar fascia after SS. As the stretching equipment used in the present study covered the toes, both the metatarsophalangeal joint and the ankle joint were dorsiflexed. A previous study showed that the elasticity of the plantar fascia is increased after 3 min of SS of toe or ankle dorsiflexion [43]. Thus, compared with the previous study [43], the SS in the present study may have resulted in an equal or greater increase in the stiffness of the plantar fascia because toe and ankle dorsiflexion were performed simultaneously. A greater muscle–tendon unit stiffness allows faster transmission of muscular force to bone, which could shorten the reaction time [44]. In addition, the plantar fascia has been shown to be one of the most important factors related to balance [45,46,47]. Thus, four sets of SS may have offset the negative (i.e., muscle strength deficits) and positive (i.e., increased plantar fascia stiffness) effects of stretching on balance, resulting in no change in balance after four sets of SS.

Unlike in previous studies [11,48], the muscle activation during the balance test did not change after SS in the present study. During the single-leg balance task, the gastrocnemius lateralis, gastrocnemius medialis, and soleus muscles are activated to maintain balance [49]. In addition, Lima et al. [11] reported an increase in the EMG amplitude of the gastrocnemius lateralis muscle during the single-leg balance task after six 45 s sets of SS. Coratella et al. [48] also reported an increased EMG amplitude during balance tasks in the vastus lateralis, biceps femoris, gastrocnemius medialis, and tibialis anterior muscles after 1800 s of SS (450 s × four exercises). Considering the positive correlation between the EMG amplitude and the COP [50], SS would have altered the neuromuscular activity during the balance task. The increased muscle activity in balance tasks after SS may be due to changes in the cerebellum, basal ganglia, ventral anterior and ventral lateral thalamus nuclei, and muscle spindles, which play an important role in maintaining balance [48]. However, in the present study, the EMG amplitude during the single-leg balance test did not change after SS. Thus, the present study suggests that short SS durations of 30–120 s do not cause changes in neuromuscular activity that affect balance.

Previous studies have shown inconsistent results regarding the effect of SS on balance, reporting improved [19,20,21,22,23,24,25,26], impaired [9,10,11,21,27], and no change [19,28,29,30,31,32] in balance, respectively. This discrepancy in results among studies may be due to differences in stretching methods, stretching sites, warm-up methods, balance assessment methods, and participant characteristics. For example, some studies that have shown improved balance after SS have evaluated balance using COP variables [21,22,23,25]. However, these studies have reported improved balance in either two-leg open-eyed standing measurements or one-leg closed-eye standing measurements [21,22,23,25]. In contrast, studies that assessed balance using the one-leg open-eyed standing measurement, as in the present study, have reported a decrease or no change in balance [11,23,30], suggesting that the balance assessment method may influence the results. Therefore, it is necessary to clarify in the future how these factors affect the change in balance after SS.

The lack of gender differences in balance changes after SS could be attributed to the lack of gender differences in the effects of SS on stiffness and muscle strength. No gender difference in balance change was found in this study, regardless of SS duration, which is consistent with the results of a previous study that found no significant gender differences in change in balance after SS in 21 men and 21 women [24]. Muscle–tendon unit stiffness is one factor that influences balance, but it has been reported that there were no gender differences in stiffness changes after SS [51]. In addition, muscle strength is another factor that influences balance, and it has been reported that there were no differences in the effects of SS on muscle strength between men and women [52]. Thus, the lack of gender differences in changes in stiffness and muscle strength after SS may account for the lack of gender differences in balance after SS in this study.

There are three limitations in this study. First, the participants were all young adults. As the change in the risk of fall associated with changes in balance is an important issue in the elderly [5], it is important to determine whether the results of the present study can be applied to the elderly. A previous study reported that there was no difference in dynamic balance changes assessed by voluntary movement of the COP between young and elderly participants after 90 s of SS of the posterior aspect of the bilateral lower legs [32]. This suggests that the present results may also be applicable to the elderly; however, further study is needed. Secondly, eight participants in this study had current exercise habits. Furthermore, no information was available regarding the past exercise history or exercise habits of any of the participants. Therefore, the subjects’ current and past exercise history and exercise habits may have affected the results. Lastly, although none of the participants in this study indicated that they habitually performed stretching, it is possible that SS was habitually performed as part of warming up or other activities in the eight participants with exercise habits. In this study, only the content of the main exercise was checked for participants with exercise habits and not the content of the warm-up, etc. If there were participants who habitually performed SS in the warm-up, etc., this may have affected the results.

## 5. Conclusions

In conclusion, the present study found that SS duration affects balance, as assessed by open-eyed single-leg standing measurements. However, there were no gender differences in SS-induced changes in balance. The present study also revealed that changes in the maximal dorsiflexion angle or passive torque after SS were not associated with balance impairment. For stretching methods similar to those used in this study, 60 s of SS should be avoided.

## Figures and Tables

**Table 1:** Stretching-induced changes in electromyography amplitude (%MVC) in the balance test.

		Gastrocnemius Medialis Muscle			Tibialis Anterior Muscle		
1 set	Pre	9.5	±	9.2	3.3	±	2.1
	Post	9.4	±	9.6	3.2	±	2.5
2 sets	Pre	8.3	±	5.3	3.0	±	1.9
	Post	8.2	±	5.4	4.0	±	2.5
4 sets	Pre	9.3	±	9.2	3.2	±	2.1
	Post	10.1	±	13	3.4	±	2.6
CON	Pre	8.4	±	5.2	2.8	±	1.6
	Post	7.3	±	5.3	3.2	±	1.9

Data are expressed as mean ± SD. CON: control.

**Table 2:** Gender differences in stretching-induced changes in standing duration (second), COP displacement (mm), COP velocity (mm/s), COP area (mm^2^), and electromyography amplitude (%MVC) in the balance test.

														EMG Amplitude					
		Standing Duration			COP Displacement			COP Velocity			COP Area			Gastrocnemius Medialis Muscle			Tibialis Anterior Muscle		
1 set																			
Pre	men	20.0	±	0.0	298.0	±	73.7	29.8	±	7.4	334.3	±	83.2	5.6	±	3.1	3.3	±	1.6
	women	20.0	±	0.0	255.6	±	57.5	25.6	±	5.7	307.6	±	167.5	13.9	±	12	3.3	±	2.7
Post	men	20.0	±	0.0	310.3	±	80.8	31.0	±	8.1	382.8	±	132.6	6.2	±	4.8	2.6	±	1.5
	women	20.0	±	0.0	258.7	±	67.1	25.9	±	6.7	306.4	±	137.1	13.0	±	13	3.8	±	3.2
2 sets																			
Pre	men	20.0	±	0.0	287.2	±	49.0	28.7	±	4.9	303.6	±	160.1	5.9	±	3.6	2.6	±	1.9
	women	20.0	±	0.0	255.4	±	71.5	25.5	±	7.1	279.4	±	159.0	10.9	±	5.8	3.4	±	2.0
Post	men	20.0	±	0.0	351.7	±	88.4	35.2	±	8.8	505.1	±	243.9	7.2	±	3.5	3.2	±	1.8
	women	19.0	±	3.6	278.4	±	80.4	27.8	±	8.0	306.0	±	125.8	9.3	±	7	4.7	±	3.0
3 sets																			
Pre	men	20.0	±	0.0	306.3	±	62.8	30.6	±	6.3	338.2	±	137.4	6.3	±	3.2	3.2	±	1.8
	women	20.0	±	0.0	251.8	±	53.6	25.2	±	5.4	286.9	±	170.4	12.7	±	12	3.3	±	2.6
Post	men	20.0	±	0.0	333.3	±	107.6	33.3	±	10.8	435.8	±	221.2	6.2	±	3.5	2.6	±	0.8
	women	20.0	±	0.0	256.4	±	82.4	25.6	±	8.2	307.6	±	151.2	14.5	±	18	4.6	±	3.7
CON																			
Pre	men	20.0	±	0.0	323.8	±	58.4	32.4	±	5.8	390.5	±	115.6	7.5	±	4.2	3.3	±	2.0
	women	20.0	±	0.0	242.6	±	64.3	24.3	±	6.4	268.7	±	98.7	9.5	±	6.2	2.3	±	0.9
Post	men	19.4	±	1.8	311.7	±	76.6	31.2	±	7.7	347.1	±	105.4	6.8	±	5.3	3.9	±	2.1
	women	20.0	±	0.0	242.4	±	68.0	24.3	±	6.8	282.4	±	131.6	7.8	±	5.5	2.3	±	1.1

Data are expressed as mean ± SD. No gender differences were found in the change in standing duration, COP displacement, COP velocity, COP area, or electromyography amplitude in balance test after SS or CON. CON: control.

**Table 3:** Stretching-induced changes in passive torque (Nm).

		Final 1°			Final 5°			Final 9°			Final 13°			Maximal Dorsiflexed Position		
1 set	Pre	10.2	±	7.2	13.0	±	9.0	16.7	±	11.0	21.7	±	13.8	21.8	±	13.7
	Post	10.5	±	8.2	13.5	±	9.8	17.1	±	12.0	21.7	±	14.6	25.0	±	16.8 *^,†^
2 sets	Pre	10.0	±	7.7	12.9	±	9.2	16.8	±	11.6	21.2	±	13.8	21.7	±	14.3
	Post	10.9	±	8.1 *	13.8	±	9.9 *	17.3	±	11.6	21.6	±	14.0	25.1	±	16.4 *
4 sets	Pre	9.6	±	6.2	12.4	±	7.8	16.1	±	9.9	20.5	±	12.3	20.7	±	12.6
	Post	10.1	±	6.4 *	13.0	±	8.1 *	16.6	±	10.0	20.9	±	12.3	25.8	±	15.4 *
CON	Pre	9.6	±	7.2	12.1	±	8.8	15.7	±	11.0	20.3	±	13.8	21.1	±	14.0
	Post	10.0	±	7.7 *	12.7	±	9.5	16.1	±	11.5	20.4	±	14.5	22.5	±	15.3

Data are expressed as mean ± SD. * Significantly different from pre (*p* < 0.05). † Significantly different from post in CON (*p* < 0.05). CON: control.

**Table 4:** Stretching-induced changes in displacement of muscle–tendon junction (mm).

		Final 1°			Final 5°			Final 9°			Final 13°			Maximal Dorsiflexed Position		
1 set	Pre	0	±	0	1.9	±	1.2	3.6	±	1.6	5.3	±	1.8	5.5	±	2.0
	Post	0	±	0	2.0	±	1.0	3.9	±	1.3	5.0	±	1.5	6.1	±	2.0
2 sets	Pre	0	±	0	2.0	±	1.0	4.0	±	1.2	5.5	±	1.6	5.6	±	1.8
	Post	0	±	0	1.6	±	1.1	3.5	±	1.3	5.1	±	1.2	6.0	±	1.8
4 sets	Pre	0	±	0	2.4	±	1.0	4.2	±	1.4	5.7	±	1.9	5.7	±	1.8
	Post	0	±	0	2.1	±	1.1	3.8	±	1.6	5.5	±	1.7	6.9	±	2.3
CON	Pre	0	±	0	2.3	±	0.9	4.1	±	0.9	5.5	±	1.4	5.8	±	1.5
	Post	0	±	0	2.2	±	1.4	3.8	±	1.6	5.4	±	1.6	6.2	±	1.8

Data are expressed as mean ± SD. Displacement of muscle–tendon junction was increased with increase in angle, except for the difference between the final 13° and the maximal dorsiflexed position in pre. CON: control.

**Table 5:** Stretching-induced changes in electromyography amplitude (%MVC) in passive dorsiflexion measurement.

		Initial Period						Final Period					
		Gastrocnemius Medialis Muscle			Tibialis Anterior Muscle			Gastrocnemius Medialis Muscle			Tibialis Anterior Muscle		
1 set	Pre	1.3	±	2.2	0.8	±	0.7	2.9	±	5.9	1.0	±	1.2
	Post	1.3	±	2.4	0.8	±	0.8	2.1	±	3.3	1.0	±	1.4
2 sets	Pre	1.3	±	2.6	1.3	±	2.0	1.7	±	2.8	1.3	±	1.9
	Post	1.3	±	2.6	1.3	±	1.8	1.8	±	2.9	1.3	±	1.8
4 sets	Pre	1.4	±	2.3	0.8	±	1.2	1.9	±	2.2	1.0	±	1.4
	Post	1.1	±	1.3	0.8	±	1.1	1.8	±	1.9	1.0	±	1.4
CON	Pre	0.9	±	0.7	0.6	±	0.6	2.0	±	2.4	0.8	±	1.4
	Post	0.9	±	0.7	0.6	±	0.7	1.7	±	1.9	0.8	±	1.0

Data are expressed as mean ± SD. CON: control.

**Table 6:** Pearson’s correlation coefficient of the relationship between the change in maximal dorsiflexion angle or passive torque after 2 sets of SS and the change in COP variables after 2 sets of SS.

	Change in COP Displacement	Change in COP Velocity	Change in COP Area
Change in ROM	*r* = −0.280 (*p* = 0.232)	*r* = −0.280 (*p* = 0.231)	*r* = 0.123 (*p* = 0.605)
Change in passive torque at			
Final 1°	*r* = 0.049 (*p* = 0.839)	*r* = 0.048 (*p* = 0.841)	*r* = 0.082 (*p* = 0.732)
Final 5°	*r* = 0.032 (*p* = 0.894)	*r* = 0.031 (*p* = 0.898)	*r* = 0.049 (*p* = 0.836)
Final 9°	*r* = −0.008 (*p* = 0.973)	*r* = −0.008 (*p* = 0.973)	*r* = 0.001 (*p* = 0.998)
Final 13°	*r* = −0.009 (*p* = 0.969)	*r* = −0.010 (*p* = 0.966)	*r* = 0.031 (*p* = 0.896)
Maximal dorsiflexed position	*r* = −0.166 (*p* = 0.485)	*r* = −0.167 (*p* = 0.483)	*r* = 0.221 (*p* = 0.350)

COP: center of pressure.

## Data Availability

Data relating to this article will be made available upon request to the corresponding author.

## Abbreviations

The following abbreviations are used in this manuscript:

SS	Static stretching
COP	Center of pressure
CON	Control
EMG	Electromyography
